# Workplace homicides committed by firearm: recent trends and narrative text analysis

**DOI:** 10.1186/s40621-019-0184-0

**Published:** 2019-03-18

**Authors:** Mitchell L. Doucette, Maria T. Bulzacchelli, Shannon Frattaroli, Cassandra K. Crifasi

**Affiliations:** 1 Injury Prevention Center, Connecticut Children’s Medical Center, Hartford, Connecticut USA; 2 Department of Health Sciences, Eastern Connecticut State University, Willimantic, CT USA; 3 Undergraduate Program in Public Health Studies, Johns Hopkins Krieger School of Arts and Sciences, Baltimore, MD USA; 4 Center for Gun Policy and Research, Johns Hopkins Bloomberg School of Public Health, Baltimore, MD USA

**Keywords:** Workplace homicide, Workplace violence, Firearm violence, Descriptive epidemiology

## Abstract

**Background:**

Firearm workplace homicides are a significant problem in the United States. We sought to provide a current, national-level examination of these crimes and examine how perpetrators accessed firearms used in workplace homicides.

**Methods:**

We abstracted information on all firearm workplace homicides from the Bureau of Labor Statistics’ Census of Fatal Occupational Injuries from 2011 to 2015. We classified deaths by perpetrator’s relationship to the workplace/victim, motive (robbery v. non-robbery), circumstance (argument v. other circumstances), and firearm access points using narrative text fields.

**Results:**

There were 1553 firearm workplace homicides during the study period. Robbery crime trended downward from 2011 to 2015. In contrast, non-robbery crimes constituted almost 50% of the homicides and trended upward in recent years. Customers and co-workers were the most frequent perpetrators of non-robbery crimes, most after an argument. While customers and co-workers who commit these crimes were often armed at the time of the argument, some were not and retrieved a firearm from an unspecified location before committing a homicide. Thus, immediate *and* ready firearm access was commonly observed in argumentative workplace deaths.

**Conclusions:**

Limiting firearm access in the workplace is a possible measure for preventing deadly workplace violence and should be considered as part of a comprehensive strategy for addressing this reemerging public health concern.

## Background

Despite reductions in workplace homicide over the past two decades, the latest data suggest that trend is reversing (Konda et al., 2014; Death on the Job, 2017; Release, 2017). In 2016, 500 workers were murdered on-the-job, the highest number since 2010 (Release, 2017). In nearly 80% of those deaths (*n* = 394) perpetrators used firearms (Release, 2017). The number of firearm workplace homicides increased 16.4% between 2014 and 2015, from 307 to 354, and increased another 11.2% between 2015 to 2016 (Release, 2017; News Release, 2016).

One approach to understanding trends in workplace homicides is a violence typology based on the perpetrator’s relationship to the workplace and victim: Type I violence refers to someone with no prior relationship to the workplace/victim; Type II violence refers to a customer or client of the workplace; Type III violence refers to a current or former employee of the workplace; and Type IV violence refers to someone with a personal relationship with the victim (Konda et al., 2014; Peek-Asa et al., 1997; Howard, 1996; Gurka et al., 2009; Tiesman et al., 2012; Hendricks et al., 2007; Gurka et al., 2012; Workplace Violence, 2001). Additional stratification of workplace homicides includes motivation (i.e. robbery vs. non-robbery) and circumstance (i.e. argument vs. other circumstance). Previous research found approximately 60% of workplace homicides are associated with a robbery (Gurka et al., 2009), and an estimated 20% of workplace homicides result from an argument (Konda et al., 2014; Moracco et al., 2000). Robbery crimes are primarily committed by people not associated with the business or victim (Type I), while non-robbery crimes are primarily committed by customers (Type II), other co-workers or work associates (Type III), and perpetrators who have a personal relationship with the victim (Type IV). Around 50% of non-robbery crimes involve an argument (Konda et al., 2014).

Over the last 6 years, from 2011 to 2016, the portion of workplace homicides committed with firearms was roughly 80% (Release, 2017). In one study analyzing workplace homicides in North Carolina from 1994 to 1998 the authors concluded that workplaces that permit employees to carry a firearm had nearly 5-times greater odds of having a workplace homicide compared to workplaces that prohibited weapons (Loomis et al., 2005). Furthermore, the odds that a customer or a worker is armed have likely increased in recent years as a majority of U.S. states have passed right-to-carry laws or have removed the need to obtain a permit to carry a concealed weapon (Webster, 2017; Webster et al., 2017). By-in-large, right-to-carry states remove discretion from authorities over who is issued a permit to carry a concealed firearm (Siegel et al., 2017). Authorities are most typically law enforcement officers. A nationally representative survey of gun owners found greater proportions of loaded handgun carrying in right-to-carry states compared to states without such laws (Rowhani-Rahbar et al., 2017). It is likely handgun carrying in right-to-carry states has also increased in the work environment, elevating firearm exposure for workers.

There are several gaps in the workplace homicide literature. First, we are unaware of any research characterizing the violence typology, motivation, and circumstance of firearm workplace homicides (Konda et al., 2014). Second, the original research characterizing workplace homicides by motivation used only North Carolina workplace deaths from 1994 to 2000 and the most recent, national-level epidemiologic investigations of workplace homicides have been subset analyses, focusing on non-robbery-related workplace homicides in the retail industry from 2003 to 2008 (Konda et al., 2014) and on the female experience of workplace homicides from 2003 to 2008 (Tiesman et al., 2012). Finally, no study has attempted to characterize how perpetrators access firearms used in workplace homicide, an important element of prevention. As firearm workplace homicides are on the rise, addressing these research gaps may provide context for potential interventions.

Thus, this study aims to: 1) provide a current, national-level count of firearm workplace homicides by motivation and violence type; 2) report select characteristics (e.g. the race/ethnicity, age, sex, and occupation of victim) of firearm homicides by motivation and violence; and 3) examine how perpetrators access firearms used in workplace homicides.

## Methods

We identified workplace homicides committed with a firearm in the U.S. from 2011 to 2015 using the Census of Fatal Occupational Injuries (CFOI) restricted data file. The CFOI is a national injury surveillance system that confirms workplace deaths via death certificates, workers’ compensation reports, police reports, media reports, Occupational Safety and Health Administration Investigation reports, and medical examiner reports. All confirmed workplace deaths require at least two independent source documents indicating the death was work-related. Law enforcement deaths were excluded from the analysis.

### Variable definitions

The CFOI uses the Occupational Injury and Illness Classification System (OIICS) to classify occupational injury death events. The OIICS coding changed starting in 2011 (Occupational Safety and Health Changes to OIICS, NAICS, and SOC, 2015) so our study period begins in 2011. For each death event, CFOI provides OIICS codes for the nature of injury, source of injury, secondary source of injury, and event or exposure. We used the OIICS source codes to define violence typology (e.g. Type I-IV workplace violence). Full OIICS code definitions for each violence typology are available in Table 1. For violence typology, OIICS source codes prioritize the perpetrator’s relationship to the workplace over their relationship with the victim where both relationships exist (i.e., a husband who kills his wife with whom he works would be considered a co-worker). We used the North American Industry Classification System (NAICS) to categorized industries into 8 categories: 1) Labor, 2) Retail, 3) Transportation, 4) Health Care, 5) Professional, 6) Education/arts, 7) Public Administration, and 8) Other. A full list of industries is provided in Table 3. We identified law enforcement deaths using NAICS code 922120.

**Table 1:** Firearm-related Workplace Homicides Violence Typology Counts, by Motivation and Circumstance, CFOI, 2011–2015

	Typology^a^					Total (*n* = 1553)	*P*-value
	Assailant unknown, criminal intent (Type I)	Customers or clients (Type II)	Co-worker or work associate (Type III)	Personal relationship (Type IV)	Unknown typology^b^		
Motivation							*P* < 0.0001
Non-robbery-motivated * n (%)*	175 (23.5)	157 (21.1)	200 (26.9)	179 (24.1)	33 (4.4)	744	
Robbery-motivated * n* (%)	592 (95.6)	-- (--)	12 (1.9)	10 (1.6)	-- (--)	619	
Unknown Motivation * n (%)* ^*c*^	54 (28.4)	17 (9)	4 (2.1)	5 (2.6)	110 (57.9)	190	
Circumstance^d^							*P* < 0.0001
Arguments * n* (%)	42 (12.2)	142 (41.3)	114 (33.1)	36 10.5)	10 (2.9)	344	
Conflicts * n* (%)	15 (6.8)	4 (1.8)	66 (30)	132 (60)	3 (1.4)	220	
Other Circumstances * n* (%)	118 (65.6)	11 (6.1)	20 (11.1)	11 (6.1)	20 (11.1)	180	

Fatal injury counts were generated by authors with restricted access to BLS CFOI microdata. Row may not add up to 100 due to rounding. Firearm-related workplace homicide defined by Occupational Injury and Illness Classification System codes as: Nature of injury/illness (1340); Event or Exposure (1111); Source of Injury/Illness (57, excluding 578); Secondary source of injury/illness (78, excluding 7813)

-- Indicates no data or data that do not meet BLS publication criteria

^a^Typology consists of the following OIICS source codes: Type I (5770, 5771, 5772, 5773, 5779); Type II (5730, 5740, 5750); Type III (5720); Type IV (5710, 5711, 5712, 5719, 5760); Unknown (5700, 5790)

^b^Unknown typology refers to cases where there was not enough information pertaining to the perpetrator to characterize violence typology

^c^Unknown motivation refers to cases where there was not enough information pertaining to what motivated the workplace homicide

^d^Circumstance coded only among cases confirmed to be non-robbery-motivated (*n* = 744)

In addition to the OIICS source codes, the CFOI restricted data file includes a narrative text field that provides a description of how the death occurred. We reviewed the narrative text fields to categorize each event’s violence typology, motivation, circumstance, and firearm access point. For Type I, if the narrative text stated the assailant was unknown, for instance in cases involving unsolved murders, the violence type was coded as ‘unknown typology.’ Else, OIICS source of injury codes were used. The author MLD coded the workplace homicides’ violence typology, motivation, and circumstance.

We coded motivation based on the existing literature. Robbery cases were deaths where robbery was the primary motivation, confirmed by police reports (Konda et al., 2014; Gurka et al., 2009). Non-robbery cases were deaths where robbery was known not to be the motive. If the narrative text stated that the motivation for the crime was uncertain, but robbery had been ruled out, we categorized the death as non-robbery. If there was no known motivation for the homicide, motivation was coded as ‘unknown motivation.’

We coded circumstance based on a modified approach used by Konda, Tiesman, Hendricks and colleagues (2014) in which non-robbery workplace homicides are *Arguments* or *Other Circumstances* based on narrative text data (Konda et al., 2014). We added a category: *Arguments*, where the narrative text stated the crime immediately followed from a verbal altercation; *Conflicts*, where it is highly likely a past or current altercation between the perpetrator and the worker existed, but a direct argument was not confirmed as part of the event’s narrative text; and *Other Circumstances*, where the worker was not killed as part of any kind of altercation, (e.g. random gun firing, caught in cross fire, a mass shooting/terrorism event). The full list of circumstances is presented in Table 4. We did not classify robberies as part of circumstance because they represent their own crime subset.

To establish firearm access points, we analyzed the narrative text data using a content analysis approach (Silvermann & Maevasti, 2008). To identify the ways perpetrators accessed firearms, author MLD sampled 20% of the cases and read their narrative text fields. We selected the sample using a random number generator in Excel to generate random integers that corresponded to event identification numbers provided by the CFOI restricted data file. Narrative text fields were read and we noted any way in which shooters accessed the firearms used in the homicide. Identified points of firearm access were then coded throughout the remaining dataset by author MLD.

### Analysis

We tabulated frequencies and conducted chi-square (χ^2^) tests for statistical independence to examine differences between select characteristics of firearm workplace homicides. We used STATA version 15 for analysis (StataCorp, 2017). The research was conducted with restricted access to Bureau of Labor Statistics data. The views expressed here do not necessarily reflect the views of the Bureau of Labor Statistics. The Johns Hopkins Bloomberg School of Public Health determined this as not human subject research.

## Results

From 2011 to 2015, there were 1727 identified firearm workplace homicides, including 174 homicides of law enforcement officers. Of the 1553 non-law enforcement firearm workplace homicides, 39.9% (*n* = 619) took place during robberies, 47.9% (*n* = 744) were not motivated by robbery, and 12.2% (*n* = 190) were unknown motivation (Table 1). Among the 744 firearm workplace homicides not motivated by robbery, 46.2% (*n* = 344) involved an argument, 29.6% (*n* = 220) involved a conflict, and 24.2% (*n* = 180) were other circumstances (Table 1).

Typology differed significantly (*p* < 0.001) between motivation and circumstance (Table 1). The majority of robbery workplace homicides (95.6%) were committed by an unknown assailant (Type I). Firearm workplace homicides not committed as part of a robbery were nearly equal across violence types. Customers (41.3%; *n* = 142) and co-workers/work associates (33.1%; *n* = 114) committed the highest percentage of the *argumentative* deaths (*n* = 344). Individuals with a personal relationship to the employee (60%; *n* = 132) and co-workers/work associates (30%; *n* = 66) committed the majority of firearm workplace homicides based in *conflict* (*n* = 220). The majority of *other circumstances* (*n* = 180) were committed by an unknown assailant (65.6%; *n* = 118).

### Characteristics of firearm workplace homicides

The number of robbery firearm workplace homicides steadily declined over the study period, down from 134 in 2011 to 112 in 2015 (Table 2**)**. This is juxtaposed against the number of non-robbery crimes, which displayed an erratic, upward trend. The number of non-robbery crimes committed by a co-worker increased from 30 in 2011 to 51 in 2015. Robbery crimes (*n* = 616) were most often committed on Mondays, Tuesdays, and Fridays (*n* = 95; 15.4% each) and most often occurred between 3:00 PM and 11:00 PM (*n* = 280; 45.5%), while non-robbery crimes (*n* = 711) were most often committed on Wednesdays (*n* = 121; 17%) and most often occurred between 7:00 AM and 3:00 PM (*n* = 312; 43.9%). Nearly the same number of white workers (*n* = 311; 50.5%) and non-white workers (*n* = 305; 49.5%) were killed as part of robbery crimes, whereas almost 1.5 times as many white workers (*n* = 424; 59.6%) compared to non-white workers (*n* = 287; 40.4%) were killed as part of a workplace homicide not motivated by robbery (Table 3**)**. For both robbery and non-robbery crimes, Hispanic workers accounted for approximately 16% of the total firearm workplace homicides (Table 3**)**.

**Table 2:** Event Characteristics of Firearm-related Workplace Homicides by Motivation and Violence Typology, CFOI 2011–2015

	Robbery-Motivated^a^		Non-robbery motivated				
	Type I	*Total* *(n = 616)*	Type I	Type II	Type III	Type IV	*Total* *(n = 711)*
Year (*n, %)*							
2011	132 (98.5)	*134*	25 (19.7)	36 (28.4)	30 (23.6)	36 (28.4)	*127*
2012	128 (95.5)	*134*	45 (27.4)	36 (22)	40 (24.4)	43 (26.2)	*164*
2013	120 (95.2)	*126*	32 (23.2)	24 (17.4)	52 (37.7)	30 (21.7)	*138*
2014	107 (97.3)	*110*	32 (26.5)	28 (23.1)	27 (22.3)	34 (28.1)	*121*
2015	105 (93.8)	*112*	41 (25.5)	33 (20.5)	51 (31.7)	36 (22.4)	*161*
Day of week (*n, %)*							
Sunday	86 (93.5)	*92*	25 (28.09)	31 (34.8)	17 (19.1)	16 (18)	*89*
Monday	94 (99)	*95*	19 (19.8)	19 (19.8)	28 (29.2)	30 (31.3)	*96*
Tuesday	90 (94.7)	*95*	26 (25.2)	20 (19.4)	25 (24.3)	32 (31.1)	*103*
Wednesday	72 (97.3)	*74*	23 (19)	25 (20.7)	45 (37.2)	28 (23.1)	*121*
Thursday	79 (97.3)	*81*	31 (26.1)	18 (15.1)	37 (31.1)	33 (27.7)	*119*
Friday	93 (97.9)	*95*	27 (25)	26 (24.1)	33 (30.6)	22 (20.4)	*108*
Saturday	78 (92.9)	*84*	24 (32)	18 (24)	15 (20)	18 (24)	*75*
Time (*n, %)*							
7:00 AM-3:00 PM	126 (98.4)	*128*	86 (27.6)	38 (12.2)	101 (32.4)	87 (27.9)	*312*
3:00 PM-11:00 PM	268 (95.7)	*280*	51 (25)	39 (19.1)	57 (27.9)	57 (27.9)	*204*
11:00 PM-7:00 AM	171 (95)	*180*	31 (19)	72 (44.2)	32 (19.6)	28 (17.2)	*163*

Fatal injury counts were generated by authors with restricted access to BLS CFOI microdata. Table excludes 146 workplace homicides without a known typology. The following characteristics contain missing information: There are 60 cases with missing time of death information

-- Indicates no data or data that do not meet BLS publication criteria

^a^Excludes 24 type II-type IV firearm-related workplace homicides

**Table 3:** Characteristics of the victims of Firearm-related Workplace Homicides by Motivation and Violence Typology, CFOI 2011–2015

	Robbery-Motivated^a^		Non-robbery motivated				
	Type I	*Total* *(n = 616)*	Type I	Type II	Type III	Type IV	*Total* *(n = 711)*
Industry^b^ (*n, %)*							
Labor	60 (93.8)	*64*	22 (16.7)	14 (10.6)	67 (50.8)	29 (22)	*132*
Retail	386 (95.6)	*404*	33 (16.3)	78 (38.4)	37 (18.2)	55 (27.1)	*203*
Transportation	85 (98.8)	*86*	18 (34.6)	12 (23.1)	11 (21.2)	11 (21.2)	*52*
Health care	5 (100)	*5*	5 (10.4)	8 (16.7)	9 (18.8)	26 (54.2)	*48*
Professional	21 (100)	*21*	10 (15.4)	18 (27.7)	20 (30.8)	17 (26.2)	*65*
Education/arts	6 (100)	*6*	13 (30.2)	9 (20.9)	11 (25.6)	10 (23.3)	*43*
Public Administration	6 (100)	*6*	45 (56.3)	0 (0.0)	32 (40)	3 (3.8)	*80*
Other	23 (95.8)	*24*	29 (33)	18 (20.5)	13 (14.8)	28 (31.8)	*88*
Gender (*n, %)*							
Female	57 (95)	*60*	29 (16)	15 (8.3)	27 (14.9)	110 (60.8)	*181*
Male	535 (96.2)	*556*	146 (27.6)	142 (26.8)	173 (32.6)	69 (13)	*530*
Age (*n, %)*							
16–19	10 (90.9)	*11*	-- (--)	-- (--)	3 (37.5)	3 (37.5)	*8*
20–24	51 (96.2)	*53*	8 (15.7)	16 (31.4)	15 (29.4)	12 (23.5)	*51*
25–34	110 (94.5)	*116*	44 (28)	34 (21.7)	44 (28)	35 (22.3)	*157*
35–44	109 (96.5)	*113*	46 (26.6)	37 (21.4)	38 (22)	52 (30.1)	*173*
45–54	158 (95.8)	*165*	37 (19.3)	42 (21.9)	59 (30.7)	54 (28.1)	*192*
55–64	112 (98.5)	*114*	26 (27.1)	19 (19.8)	32 (33.3)	19 (19.8)	*96*
65+	42 (95.5)	*44*	13 (38.2)	8 (23.5)	9 (26.5)	4 (11.8)	*34*
Race (*n, %)*							
White	298 (95.8)	*311*	118 (27.8)	75 (17.7)	128 (30.2)	103 (24.3)	*424*
Non-White	294 (96.4)	*305*	57 (19.9)	82 (28.6)	72 (25.1)	76 (26.5)	*287*
Ethnicity (*n, %)*							
Hispanic	98 (99)	*99*	27 (21.6)	30 (24)	36 (28.8)	32 (25.6)	*125*
Non-Hispanic	493 (95.5)	*516*	147 (25.3)	126 (12.7)	163 (28.1)	144 (24.8)	*580*

Fatal injury counts were generated by authors with restricted access to BLS CFOI microdata. Table excludes 146 workplace homicides without a known typology. The following characteristics contain missing information: There are 7 cases with missing ethnicity information

-- Indicates no data or data that do not meet BLS publication criteria

^a^Excludes 24 type II-type IV firearm-related workplace homicides

^b^Industry was determined by combined North American Industry Classification System codes. Labor industry consists of Agriculture, Mining, Utilities, Construction, Manufacturing, Wholesale, and Waste Management. Retail industry consist of Retail and Accommodations/food services. Professional industries consist of Finance/insurance, Information, Real Estate, Professional Scientific Services, Managers. All other categories are self-contained NAICS codes

Of the 616 crimes motivated by robbery, 404 (65.6%) were committed in the retail industry (Table 3**)**. This percent is more than two times higher than the percent of retail-related crimes among non-robbery workplace homicides (28.5%; *n* = 203). For non-robbery crimes in the retail industry, most were committed either by a customer (*n* = 78; 38.4%) or someone the victim knew (*n* = 55; 27.1%). For non-robbery firearm workplace homicides committed in the labor industries (*n* = 132), which include construction, mining, and agriculture (see Table 3 for full list), co-workers committed most (*n* = 67; 50.8%) crimes.

### Circumstance

Nearly half of the firearm workplace homicides not motivated by robbery (*n* = 744) were associated with arguments (*n* = 344) (Table 4). Within the *arguments* category, the highest percentage (*n* = 165; 22.2%) was unknown origin, where the CFOI narrative does not discuss how the altercation began, followed by job-related arguments (*n* = 47; 6.3%), which includes disputes over work hours, being fired, or work conditions. Among the *conflict* category, the highest percentage of non-robbery firearm workplace homicides was due to personal relationships (*n* = 136; 18.3%), followed by coworker/ex-coworker deaths of unknown circumstance (*n* = 64; 8.6%). Of the 136 conflict deaths related to personal relationships, 103 were female workers (75.7%) (data not shown). Of the 103 female workers killed in a conflict related to a personal relationship, 93 (90%) were killed by a known assailant (Type IV violence), typically a domestic partner (data not shown). Among the *other circumstances* category (*n* = 180), employees were most commonly killed as part of mass shooting events, including terrorist attacks (*n* = 68; 9.1%).

**Table 4:** Firearm-related workplace homicides by circumstance among non-robbery-motivated crimes, CFOI, 2011–2015

Circumstance	N	%
Arguments		
Asked to leave establishment	23	3.1
Breaking up a fight	23	3.1
Job related (work hours, employee fired, work conditions)	47	6.3
Denied access to establishment	13	1.8
Over personal relationship	13	1.8
Over sale of merchandise	24	3.2
Escorting unruly patrons	–	–
Refused service	–	–
Arguments, other/unknown	165	22.2
Disgruntled customer	31	4.2
*Total*	*344*	*46.2*
Conflicts		
Personal relation, unknown circumstance	136	18.3
Coworker/ex-coworker, unknown circumstance	64	8.6
Act of revenge	20	2.7
*Total*	*220*	*29.6*
Other Circumstances		
Random gun firing	12	1.6
Caught in crossfire	15	2
Trying to get away (suspect)	4	0.5
Legal intervention	8	1.1
Active shooter respondent	4	0.5
Intervening in situation (civilian)	13	1.8
Gang related	6	0.8
Mass shooting/terrorism/shooting rampage	68	9.1
Drug deal	4	0.5
Unknown, robbery ruled out	19	2.6
Other	27	3.6
*Total*	*180*	*23.6*
Total	744	100

Fatal injury counts were generated by authors with restricted access to BLS CFOI microdata. Table include 744 non-robbery-motivated workplace homicides

-- Indicates no data or data that do not meet BLS publication criteria

Percent may not add up to 100 due to rounding

### Firearm access points of workplace homicides

The content analysis of narrative text fields revealed 6 ways perpetrators accessed firearms: 1) on-person, 2) from a home, 3) from a car, 4) from a location within work (such as an office or locker), 5) stolen from victim, or 6) retrieved in an unspecified way. Overall, there were 292 firearm workplace homicides for which perpetrator firearm access could be determined (Table 5). Of the 292 deaths, violence typology was unable in 15 cases. These crimes most commonly committed by customers (*n* = 120; 43.3%) followed by co-workers or work associates (*n* = 71; 25.6%) (Table 5**)**. Customers most commonly had the firearm on their person (*n* = 76; 63.3%), retrieved the firearm in an unspecified way (*n* = 28; 23.3%), or retrieved the firearm from their car (*n* = 11; 9.2%). Similarly, co-workers most commonly had the firearm on their person (*n* = 39; 54.9%), retrieved the firearm used in an unspecified way (*n* = 19; 26.8%), or specifically retrieved the firearm used from their car while at work (*n* = 9; 12.7%).

**Table 5:** Counts of firearm access points by typology, CFOI, 2011–2015^a^

	Typology				
	(Type I)	(Type II)	(Type III)	(Type IV)	*Total*
Firearm Access point					
On-person	40	76	39	23	*178*
Retrieved, Unspecified	9	28	19	4	*60*
From Car	–	11	9	–	*20*
From Home	–	4	3	–	*8*
Stolen by perpetrator	7	–	–	–	*8*
From another location	–	–	–	–	*3*
*Total*	*57*	*120*	*71*	*29*	*277*

Fatal injury counts were generated by authors with restricted access to BLS CFOI microdata. Table is firearm-related workplace homicides with a known firearm access point among argumentative deaths

-- Indicates no data or data that do not meet BLS publication criteria

^a^Table consists of only firearm-related workplace homicides with a firearm access point that was able to be determined. Excluded are 15 firearm-related workplace homicides with no known violence typology

Notably, cases for which the narrative text contained sufficient detail to discern how the perpetrators accessed their firearms were most often arguments. Argumentative firearm workplace homicides constituted 233 of the 292 cases (79.9%) (data not shown). From 2011 to 2015, 73 customers argued with an employee and then committed a workplace homicide with a firearm on their person; 39 customers argued with an employee, left to retrieve a firearm, and then returned and committed a workplace homicide. Thirty-four employees argued with another employee and then committed a workplace homicide with a firearm on their person. There were 8 instances where an employee had their own weapon used against them, with 7 of the homicides committed by an unknown assailant (Type I violence). Twenty-seven employees got into an argument with another employee, left the workplace to retrieve a firearm, and then returned to commit a workplace homicide (data not shown).

## Discussion

This manuscript describes firearm workplace homicides by violence typology, motivation, and circumstance from 2011 to 2015 nationwide. The findings here are consistent with existing literature that documents the typology of workplace homicide incidents varies by motivation and circumstance (Konda et al., 2014; Gurka et al., 2009; Moracco et al., 2000). However, unlike the prior contributions, this study indicates that non-robbery crimes now account for almost 50% of workplace homicides. This is a departure from previous estimates that found non-robbery crimes accounted for approximately one-third of workplace homicides (Konda et al., 2014; Gurka et al., 2009; Moracco et al., 2000; Loomis et al., 2002). Moreover, examining the yearly trends of robbery versus non-robbery crimes shows a steady downward trend of robbery crimes versus an erratic, upward trend in non-robbery crimes. This suggests the recent increase in workplace homicides is driven by non-robbery crimes.

Consistent with previous literature, we found that arguments were the most common circumstance among non-robbery workplace homicides (Konda et al., 2014; Moracco et al., 2000), and that customer-employee (Type II) and employee-employee (Type III) altercations constitute a large portion of argumentative workplace homicides, particularly in the retail industry (Konda et al., 2014). This paper further contextualizes these relationships. Customers and employees either accessed their firearm directly on their person or retrieved their firearm from another location. Thus, among the firearm workplace homicides for which firearm access points could be categorized, immediate as well as nearby firearm access appeared to play a role in escalating arguments into workplace homicides, particularly for customers and employees. This finding supports research from Loomis and colleagues (2005) that employee firearm access at work may lead to increased odds of a workplace homicide, and speaks to the important role firearm exposure may play in workplace deaths (Loomis et al., 2005).

An increase in firearm exposure within the general public may be partially responsible for this change. From the mid 1990’s to now many states have loosened laws regarding who can carry a concealed firearm, or eliminated oversight altogether (Webster et al., 2017; Siegel et al., 2017). States that have changed their laws in recent years have higher proportions of loaded handgun carrying than states that did not adopt such laws (Rowhani-Rahbar et al., 2017). Increased handgun carrying likely affects firearm exposure for employees, as more customers or fellow employees may be armed or have nearby access to a firearm. The increase in non-robbery firearm workplace homicides may be at least partially attributable to increases in firearm exposure in the workplace. Causal examinations of policies that increase firearm exposure are needed to assess this claim.

Restricting customer and employee firearm access in the workplace could reduce argumentative workplace homicides. Employers’ rationale for allowing firearms in their workplace is not known, but protection is a likely motivation. Findings from this research offer a direct counterpoint. Allowing customers or employees to carry firearms may lead to fatal outcomes for disagreements that otherwise might not have turned deadly. Previous literature noted de-escalation training for employees as a possible prevention strategy for reducing argumentative workplace homicides (Konda et al., 2014). This type of training includes teaching employees to identify warning signs of aggression and how to calm agitated individuals (Anderson & Clarke, 1996). These prevention strategies have proven efficacious in the health care setting (Martinez, 2016) but have not been widely examined in the general workforce. It is important to note that, as CFOI contains only workplace deaths, the number of workplace homicides prevented by an employee having a firearm is unknown and should be considered. However, we identified 8 cases in which an armed employee had their own weapon used against them.

This research presents a new way of considering workplace homicide circumstances. As the number of non-robbery workplace homicides have increased over the years, it is important to fully characterize these deaths. We offer an alternative to the existing classification structure presented by Konda and colleagues (2014) (Konda et al., 2014), adding *conflicts* to the existing categories of *arguments* and *other circumstances*. We believe it is important to consider *conflicts* separately as these types of workplace homicides are interpersonal in nature yet lack enough detail in the narrative text to conclude they stemmed from an observable argument. Moving forward, categorizing workplace homicides into *arguments, conflicts,* and *other circumstances* will help researchers and policy makers develop targeted prevention efforts.

Further, this paper offers the first accounting of the number of workers who died from a terrorist or spree-based mass shooting. A previous examination of non-robbery workplace homicides, conducted using data from 2003 to 2008, did not include mass shootings as a death circumstance (Konda et al., 2014). As public health officials develop preventive policies and interventions to stem the recent rise in mass shootings in the U.S. (Cohen et al., 2014), worker safety and health should be considered.

We could assess firearm access points in 292 of the 1553 firearm workplace homicides. Homicide incidents with sufficient narrative text to assess how perpetrators accessed their firearms were largely arguments. Argumentative deaths may have created additional sources of information for investigators to assess how perpetrators accessed their firearms (i.e., witnesses, security footage). This additional information may have allowed for richer narrative text about the incident compared to other types of workplace homicides, explaining why almost 80% of the death events with sufficient narrative detail to determine firearm access points were based in an argument.

### Limitations

CFOI is a well-established, national surveillance system that provides the most comprehensive counts of workplace deaths. However, CFOI is not without limitations. First, CFOI’s Restricted Data File does not contain state identifiers so we were unable to examine within-state trends. We were unable to assign typology in 146 (9.4%) of the 1553 deaths due to insufficient narrative text data, though this percentage is lower than has been previously reported by Gurka and colleagues (2009) (18% unknown typology) and Tiesman and colleagues (2012) (16% unknown typology) (Gurka et al., 2009; Tiesman et al., 2012). The effect of these unknown homicides on the frequencies presented here is unknown. As such, the frequencies generated may not be representative of the true firearm workplace homicide incidence from 2011 to 2015. Further, as this study does not provide rates of firearm-related workplace homicide, it is possible the upward trend in non-robbery motivated workplace homicides is explained through an increase in the overall workplace participation rate. However, an increase in workplace participation would not fully explain the downward trend of robbery motivated crimes, suggesting a minimal limitation. While we used a systematic approach, and relied on existing literature to classify motive, circumstance, typology, and firearm access points, misclassification may have occurred. To reduce the likelihood of misclassification, our methods mirrored those of past research using the CFOI Restricted Data File (Konda et al., 2014; Tiesman et al., 2012; Fayard, 2008). It is important to note that a limitation of this study, and all studies that utilize the CFOI Restricted Data file, is the difficulty faced when trying to categorized events into mutually exclusive categories based on, at times, very brief descriptions of the events. This study likely underrepresents the true impact of firearm violence as CFOI data does not contain information for non-workers. As the CFOI pertains only to deaths, no data on protective uses of firearms were available and are unknown; data pertaining to the burden of nonfatal workplace firearm violence was also missing in our data. Important to note, CFOI does not contain information on the total number of employees exposed to firearms while at work. Further, as firearm violence at work continues to be a public health issue, the CFOI should consider adopting new protocols to better understand how perpetrators access firearms, and to capture firearm exposure in general. A larger emphasis on understanding the circumstances around firearm violence within the workplace will inform future prevention efforts.

## Conclusion

This paper presents the first national-level epidemiologic investigation of firearm workplace homicides. Workplace homicides not committed as part of a robbery now account for almost 50% of total firearm workplace homicides. Moreover, robbery workplace homicides declined from 2011 to 2015. Customers and co-workers are the most frequent perpetrators of non-robbery crimes, most often as part of arguments. While customers and co-workers who commit these crimes are often armed at the time of the argument, many are not and retrieve a firearm from an unspecified location before committing a workplace homicide. Immediate and nearby firearm access plays a large role in argumentative workplace deaths. Limiting firearm access in the workplace is one possible way to prevent firearm workplace homicides and should be considered as part of a comprehensive strategy for preventing deadly workplace violence.

## Authors’ information

This manuscript represents aspects of author MLD’s Doctoral Thesis completed May 2018 at the Johns Hopkins Bloomberg School of Public Health. MLD is now a Research Scientist at the Connecticut Children’s Medical Center’s Injury Research Center and an Assistant Professor at Eastern Connecticut State University.
